# Single molecule tracking reveals functions for RarA at replication forks but also independently from replication during DNA repair in *Bacillus subtilis*

**DOI:** 10.1038/s41598-018-38289-6

**Published:** 2019-02-13

**Authors:** Hector Romero, Thomas C. Rösch, Rogelio Hernández-Tamayo, Daniella Lucena, Silvia Ayora, Juan C. Alonso, Peter L. Graumann

**Affiliations:** 1 SYNMIKRO, LOEWE-Zentrum für Synthetische Mikrobiologie, Philipps-Universität Marburg, Hans-Meerwein-Straße, Mehrzweckgebäude, 35043 Marburg, Germany; 2 Fachbereich Chemie, Philipps-Universität Marburg, Hans-Meerwein-Straße 4, 35032 Marburg, Germany; 3 Department Microbial Biotechnology, Centro Nacional de Biotecnología, CNB-CSIC, 3 Darwin St., 28049 Cantoblanco, Madrid Spain

## Abstract

RarA is a widely conserved protein proposed to be involved in recombination-dependent replication. We present a cell biological approach to identify functional connections between RarA and other proteins using single molecule tracking. We found that 50% of RarA molecules were static, mostly close to replication forks and likely DNA-bound, while the remaining fraction was highly dynamic throughout the cells. RarA alternated between static and dynamic states. Exposure to H_2_O_2_ increased the fraction of dynamic molecules, but not treatment with mitomycin C or with methyl methanesulfonate, which was exacerbated by the absence of RecJ, RecD2, RecS and RecU proteins. The ratio between static and dynamic RarA also changed in replication temperature-sensitive mutants, but in opposite manners, dependent upon inhibition of DnaB or of DnaC (pre)primosomal proteins, revealing an intricate function related to DNA replication restart. RarA likely acts in the context of collapsed replication forks, as well as in conjunction with a network of proteins that affect the activity of the RecA recombinase. Our novel approach reveals intricate interactions of RarA, and is widely applicable for *in vivo* protein studies, to underpin genetic or biochemical connections, and is especially helpful for investigating proteins whose absence does not lead to any detectable phenotype.

## Introduction

All cells devote a considerable fraction of their genome to functions that maintain genome integrity, i.e. DNA repair proteins^1–3^. Some repair pathways are error-free, such as nucleotide and base excision repair, mismatch repair, alkylation damage response, and homologous recombination (HR)^4,5^, while pathways that circumvent DNA damage, such as DNA damage tolerance and non-homologous end joining, are error prone, yet ensure progression of the cell cycle^6^. To safeguard the maintenance of efficient replication, cells contain a complex factory of different proteins working together at replication forks, including not only replication but also recombination and DNA repair proteins^7–9^.

During exponential growth, HR is the main pathway used by cells to repair DSBs. Additionally, HR is also involved in the repair of lesions that produce a block of the replication forks. HR occurs in a cascade of events^1,2^: RecA is the central player of HR, and the different accessory factors that assist RecA can be divided into four broad classes: those that act before homology search (end resection [AddAB or RecJ-RecQ(RecS)-SsbA] and RecA mediators [RecO-RecR and SsbA]), those that act during homology search and DNA strand exchange (known as RecA modulators: RecF, RecX, RecU]), those that act after DNA strand exchange (involved in processing of recombination intermediates [RadA, RecG, RuvAB, RecU, RecQ(RecS)-TopIII-SsbA]), and finally proteins with a poorly understood role, like RadA (Sms) or RecD2^10–13^. At collapsed forks (one-ended DSBs) or at two-ended DSBs, RecN is recruited amongst the first proteins, and plays an important role in the assembly of a repair centre^14,15^. The DNA ends are resected by the AddAB complex or by RecJ, which acts in concert with a RecQ-like helicase (RecQ or RecS)^16^. RecA-loading and filament formation are regulated by accessory factors including mediators, i.e. RecO and RecR, and modulators RecF, RecX, and RecU^17–21^. After homology search during canonical DSB repair, Holliday junction (HJ) structures are formed that are processed by RecG, RuvAB or RadA/Sms DNA helicases and resolved by RecU or dissolved by RecQ-TopoIII^10–12^.

In this work, we have studied RarA (Replication-Associated Recombination protein A), also named MgsA, first described by David Sherratt’s laboratory as a replication-associated protein^22^. RarA has low sequence identity with RuvB and DnaX of 26% and 24% in *E*. *coli*; and 29% and 24% in *B*. *subtilis* (Fig. S1)^22^. The RarA protein family is ubiquitous and conserved from bacteria to human. *B*. *subtilis* RarA (originally termed YrvN) shares identity with *E*. *coli* RarA, budding yeast Mgs1 and mammalian Werner helicase-interacting protein 1 (WRNIP1/WHIP) (Fig. S1). The structure of *E*. *coli* RarA comprises three apparent domains: the ATPase and the tetramerization domain, which are conserved to *B*. *subtilis* RarA, in contrast to the helicase lid domain. Furthermore, both N-terminal and C-terminal ends carry several changes in different residues between *B*. *subtilis* and *E*. *coli* RarA^23^. Eukaryotic homologs have an additional ubiquitin-binding Zn finger N-terminal domain, which is not present in the prokaryotic proteins.

Although several studies agreed with the idea that RarA acts in both replication and recombination processes, which is supported by a recent genetic study^24–28^, its function is still unknown. *E*. *coli* RarA genetically interacts with SeqA^29^, RecQ^24^, UvrD^26^ or RecA^26^ and may act at blocked forks in certain replication mutants (e.g., DnaE^ts^)^25,26^. *In vitro*, *E*. *coli* RarA interacts with SSB protein, and it may separate the DNA strand at the end of the duplex to produce the entry of the replicative hexameric DnaB DNA helicase, supporting the idea that RarA acts at replication forks^27^. Much less is known about RarA in other bacteria.

The *B*. *subtilis rarA* gene, which is monocistronic, is constitutively expressed, but its expression is markedly enhanced by stressors such as diamide, ethanol, high salt or H_2_O_2_^30^. RarA interacts with SsbA, which in turn interacts with recombination (RecQ, RecS, RecJ, RecG, RecO, RecD2) and replication (PriA, DnaG, DnaE) proteins^31^. *In vitro*, *B*. *subtilis* RarA interacts with SsbA, and modulates initiation of PriA-dependent DNA replication^28^. In budding yeast, Mgs1 is proposed to be part of an alternative DNA damage tolerance pathway for rescuing blocked replication forks, probably enhancing processivity and/or fidelity of the DNA polymerase δ, and partially overlapping with functions of the helicases Sgs1 and Srs2 in genome stability^32^. In humans, it is known that WRNIP1/WHIP physically interacts with WRN, a RecQ-like helicase^33,34^. One common point of RarA studies is the complex scenario required to produce a clear phenotype that explains all observations.

In this study, we use a novel approach to characterize proteins of unknown function, complementing genetic and biochemical approaches. We have used single molecule tracking (SMT) to monitor changes in protein dynamics of RarA in the absence of many proteins involved in DNA repair and in replication. This comprehensive study reveals strong connections of RarA with the recombination and replication machinery, and further identifies novel connections between RarA and replication and recombination proteins, many of which affect RecA activities. We also provide a novel tool to study the movement of single molecules with regard to distinct points in the cell, in this case movement of RarA relative to replication forks.

## Materials and Methods

### Bacterial strains and survival assays

*B*. *subtilis* BG214 and its isogenic derivatives are listed in Table S1^35^. Methyl methanesulfonate (MMS), H_2_O_2_ and mitomycin C (MMC) were obtained from Sigma Aldrich (Germany). Otherwise indicated the cells were grown and plated in LB medium and agar plates were grown at 37 °C. Acute and chronic viability assays were performed as previously described^35,36^.

### Epifluorescence microscopy

*B*. *subtilis* cells dilutions were grown at 25 °C in S7_50_ minimal medium to OD_600_ ~0.3. Cells were either treated with 1 mM or 0.5 mM H_2_O_2_, or with 50 ng/ml MMC, or remained untreated, and 2.5 μl of culture was spotted on cover glasses and immobilized with coverslips coated with fresh agarose 1% (w/v) in S7_50_ medium. Epifluorescence microscopy was performed using a Zeiss Axio Imager A1 microscope equipped with a 1.45 objective and an EVOLVE EMCDD camera (Photometrics). A 515 nm LED laser was used for YFP/mVenus detection, a 445 nm laser was used for CFP detection and a Xenon lamp and DAPI filter were used DAPI stained cells images when needed. Time-lapse images of YFP were taken in a maximum of 1 min, and the length of acquisition in a sample was limited to 20 min to avoid the heating of the sample. Picture acquisition was done using VisiView (2.1.2).

For the colocalization of RarA-mVenus with the replication forks, images were taken as described above and processed equally (background subtraction and Gaussian blur) using ImageJ (National Institutes of Health, Bethesda, MD) prior to the merging.

### Single-molecule tracking (SMT)

To reduce the background, cover glasses were cleaned using Hellmanex II 2% (v/v) and sonicated for at least 15 min, washed with miliQ water and dried with sterile air spray duster before mounting the cells onto the medium-containing agarose pad.

SMT was performed using a Nikon microscope equipped with an A = 1.49 objective and an Image-EM CDD camera (Hamamatsu). The central part of a 514 nm laser diode was used for mVenus detection and a Xenon/Mercury lamp was used for CFP imaging in case of dual-labelled cells. Laser light intensity was generally limited to no more than 160 W cm^−2^ on the specimen, and acquisitions were limited to 5000 frames (10 seconds) to reduce the risk of generating artefacts via heating of cells. Picture acquisition was done using VisiView (2.1.2, Visitron, Munich). For movie acquisition, a bright field image was taken to determine the shape of the cells. When required, CFP images in 200 ms exposure time were taken for additional tagged proteins using CFP. Time-lapse images were prepared with ImageJ (National Institutes of Health, Bethesda, MD) and tracks were obtained using U-track (Laboratory for Computational Cell Biology, Harvard Medical School, Massachusetts, USA). Tracks were exported together with the shape of the cell generated with MicrobeTracker (Microbial Sciences Institute, Yale, USA) to SMTracker^37^. All U-track, MicrobeTracker and SMTracker are software running in MATLAB (Mathworks). SMTracker automatically calculates: i) the distribution of the tracks in the cells with overlapping with bright field or any other signal (CFP); ii) apparent diffusion coefficient (D) and population densities based on Gaussian fit to a step-size distribution with its statistical differences based in Z-test; iii) heat maps with the preferential location of the tracks in normalized cells (sorted automatically by size into small, medium and long based on the data).

As the apparent diffusion coefficient D has some fluctuations in the different backgrounds and conditions, we defined a new parameter, the dynamic population difference (DPD), to describe the effect of the absence of one protein compared to wild type, or before and after DNA damage treatment in the same D conditions, and provide a visual view of these effect allowing fast comparison. Although in the concrete case of RarA, it is reasonable to expect that it is the static, and not the dynamic population, that represents active (because DNA-bound) RarA, we find DPD to be visually clearer than SPD (static population difference) and they are complementary.

## Results

### Functionality of the RarA-YFP construct

**Figure 1:**
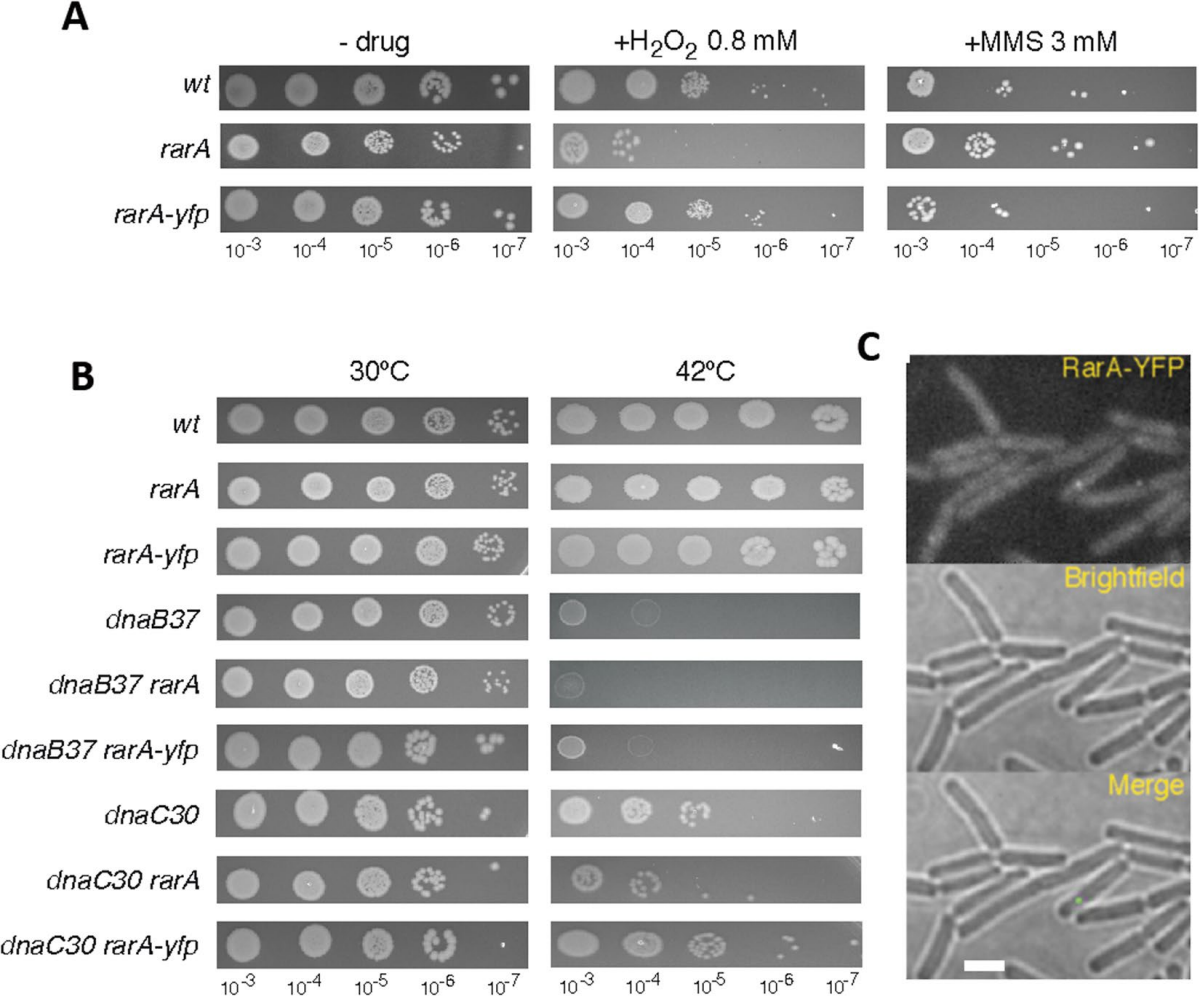
RarA-YFP characterization (**A**) Chronic viability assay for the RarA-YFP expressing strain. The presence of the YFP tag does not affect the viability of the strain; (**B**) chronic viability of ∆*recX*/RarA-YFP compared to wild type and ∆*recX* mutant. The chronic exposure to MMS produces the death of both mutant strains in the same way while wild type is still surviving; (**C**) Thermosensitivity assays for *dnaB*37 and *dnaC*30 replication mutants expressing RarA-YFP. The fluorescence tag does not affect the response of any of the single mutants; (**D**) RarA-YFP foci in *B*. *subtilis* BG1331 (*rarA-yfp*) cells during exponential growth after 700 ms exposure to 515 nm laser excitation. Only ~15% of the cells contain foci.

Traditionally, interactions of proteins are investigated by genetic (e.g. synthetic lethal screens, two hybrid screens, etc.) and biochemical means (e.g. pull-down assays, protein cross-linking, etc.). We sought to investigate the intracellular dynamics of RarA in response to different kinds of DNA damage, using RarA-YFP or RarA-mVenus (brighter variant of YFP) constructs, and analysed if its mobility is altered in different genetic backgrounds. Two strains were constructed expressing RarA-YFP or RarA-mVenus from the original gene locus, as sole source of the protein in the cell (Table S1). For testing the functionality of the fusion protein, cells were exposed to 0.8 mM (Fig. 1A) or to different doses of H_2_O_2_ for 15 min (Fig. S2A) and were plated in growth medium. RarA-YFP expressing cells showed no apparent phenotype for this drug, while null *rarA* mutant (∆*rarA*) cells were highly sensitive to the drug (Figs 1A and S2A). Likewise, RarA-mVenus expressing cells did not show sensitivity to Mitomycin C (MMC) (Fig. S2B), and RarA-YFP expressing cells were insensitive to exposure of methyl methanesulfonate (MMS) (Fig. 1A), nor were they temperature sensitive (Fig. 1B), unlike *dnaB*ts or *dnaC*ts (temperature sensitive) mutant cells. To test if the RarA-YFP fusion is also functional in replication mutants, they were tested in the above mentioned replication mutants expressing RarA-YFP grown under non-permissive conditions (thermosensitivity assays) (Fig. 1B). In all cases, the RarA-YFP construct had the same phenotype as the mutant strains not carrying the fusion, clearly showing the functionality of the fusion under all the conditions used in this work.

Once the functionality of the protein was verified, we introduced the RarA-YFP fusion into 13 recombination-deficient mutants (*∆recA*, *∆recO*, *recF15*, *∆addAB*, *∆recJ*, *∆recQ*, *∆recS*, *∆recU*, ∆*recG*, *∆ruvAB*, ∆*radA*, *∆recX* and *∆recD2*), into two Y-polymerases mutants (*∆polY1* and ∆*polY2*) related to DNA damage tolerance, and into several replication thermosensitive mutants, of which only *dnaB*37 and *dnaC*30 strains revealed a clear phenotype (see below).

### RarA forms foci in the presence of DNA damage

**Figure 2:**
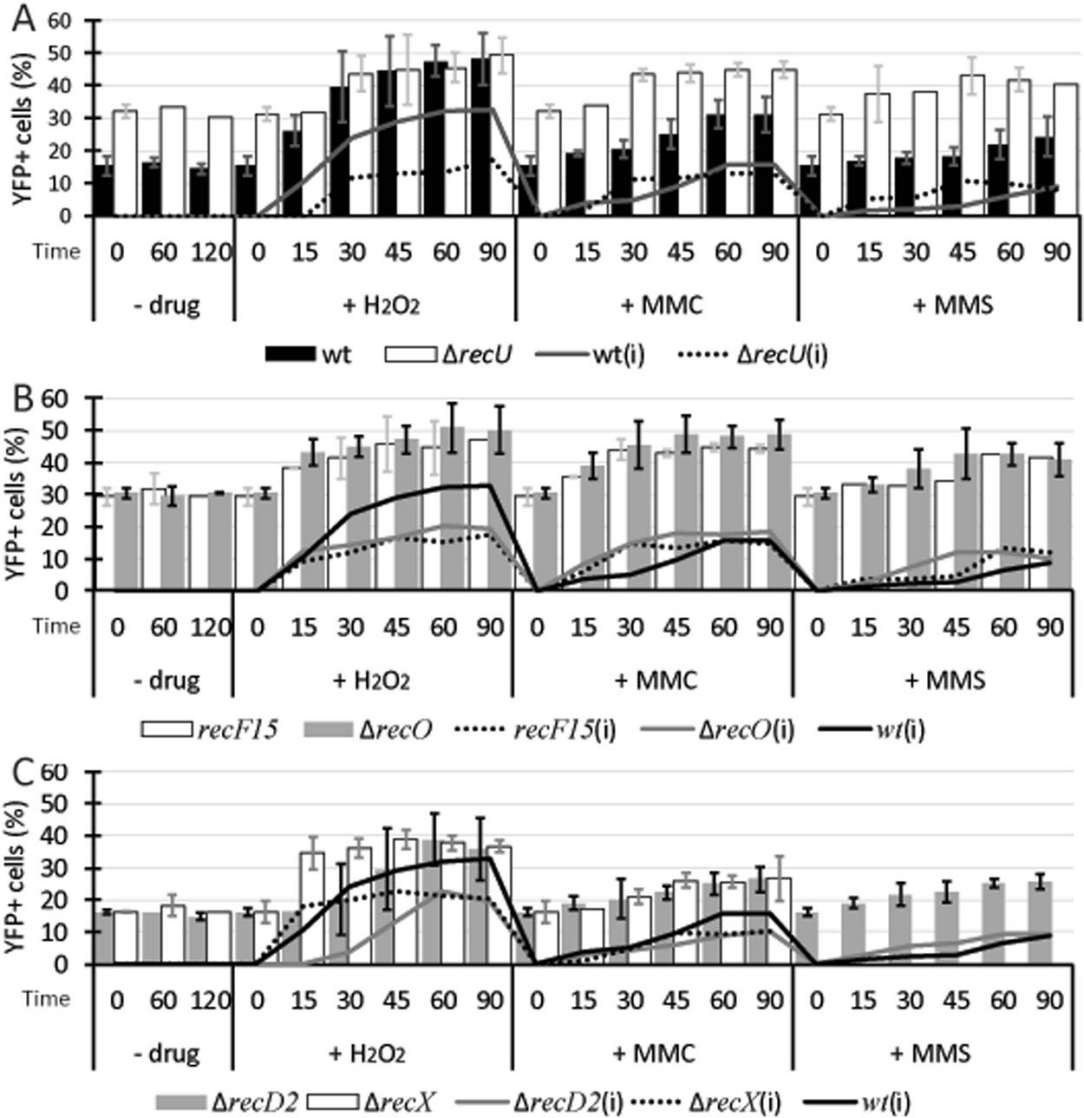
Epifluorescence for RarA-YFP in wild type cells and different recombination deficient backgrounds after DNA damage. (**A–C**) Percentage of cells that contains foci in exponential growth, and after addition of H_2_O_2_ (1 mM), MMC (50 ng/ml) or MMS (5 mM) for 60 minutes, in wild type and *∆recU* (**A**), *∆recO* and *recF15* (**B**), *∆recD2* and *∆recX* (**C**) backgrounds. Lines correspond to the increase of YFP^+^ cells considering entry into exponential growth (OD_600_ = ~0.3) as time 0. Error bars represent the standard deviation of at least three independent experiments.

RarA-YFP was visualized in wild type cells by wide field epifluorescence microscopy. In exponentially growing cells at 25 °C (OD_600_ = ~0.3), we observed that 15% of the cells contained a single RarA-YFP focus (Fig. 1C). This percentage remained apparently constant at different time points (60 and 120 min), indicating that focus formation during unperturbed growth is maintained at about a constant rate. When cells were exposed to a DNA damaging agent, the population of cells containing RarA-YFP foci increased after some time. Cells were exposed to different drugs (MMS, MMC and H_2_O_2_) to compare the responses. After addition of 5 mM MMS or 50 ng/ml of MMC, cells showed a similar type of response, starting at 30 min and reaching a plateau at 60 min with a maximum value that remained constant at least until 90 min (Fig. 2A). The intensity of the response, considered as the increase of the percentage of cells containing RarA-YFP foci, was ~15% higher after MMC and ~6% after MMS addition (Fig. 2A, grey shade). On the other hand, H_2_O_2_ addition produced an increase in the population of cells containing foci that occurred before (15 min), and had a higher maximum value (~45%) compared with MMS or MMC treatment. It is likely that RarA-YFP contributes to the repair of H_2_O_2_-induced lesions, and to a minor extent to MMC-induced lesions, while it likely does not contribute to repair MMS-induced damage see^35^. In epifluorescence, an accumulation of fluorescent molecules is needed for detection, so it is reasonable to say that in response to drugs that produce DNA damage, RarA is recruited to some position(s) within the cell in more cells than under exponential growth conditions. The presence of foci in 15% of cells grown under unperturbed conditions suggests that RarA plays a role during the cell cycle, too, at least in a fraction of cells, or during shorter periods in all cells, involving assembly of several molecules at distinct subcellular sites.

### RarA-YFP focus formation is influenced by the absence of RecA accessory proteins

The formation of RarA foci was tested in the absence of RecA mediators (RecO) or modulators (RecF, RecX, RecU) and in the absence of RecD2, which seems to be associated also with the replication forks^31^. As revealed in Fig. 2A–C, we observed an increase (twice of that seen in wild type cells) in the cells containing foci even during normal growth conditions. There were no considerable differences in the response to H_2_O_2_ and MMC, as both started at 15 min and reached a maximum between 45– 60 min. This means that the response to MMC occurred faster than in wild type cells (10% difference in 15, 30 and 45 min) but with the same increase in cells containing RarA-YFP foci. In contrast, when RecD2 was not present there was a delay in the H_2_O_2_ response (Fig. 2C) and the increase in the plateau was less pronounced than in the wild type (~20% for H_2_O_2_ and 10% for MMC, ~10% and ~5% less than wild type respectively). Also, *∆recX* cells (Fig. 2C) show similar dynamics than wild type cells but the increase in the plateau is similar to that seen in *∆recD2* cells.

### RarA foci are dynamic

The finding that RarA-YFP foci are only found in a subset of cells could indicate that it assembles only in response to circumstances that occur in a fraction of cells. It is also possible that RarA-YFP foci assemble and disassemble within a short time frame, such that at any given time, they are present in a minority of cells, although all cells do contain foci at different time points of the cell cycle. This was observed, e.g. for DNA gyrase and for topoisomerase I, which form foci within less than 1 min time scales^38^. To test these ideas, we performed time lapse experiments using RarA-mVenus because of its increased brightness compared with RarA-YFP, capturing RarA foci in 3 minutes time intervals. Supplementary Movie S1 shows that RarA-mVenus foci did not remain at the same position between 3 min intervals, but frequently moved to far away places within the cells. While 82% of the cells (108 cells analysed) did not show any foci during the 60 min duration of the experiments, 2% showed foci that disappeared are new foci that appeared at a different subcellular site, and 16% contained a single mobile focus for an extended period of time (sometimes entire 60 minutes). These data show that a) the culture is split into cells containing visible foci and cells that do not, b) that foci rarely appear and dissociate, and c) RarA assemblies can exist for a large part of the cell cycle. Bearing in mind that epifluorescence visualizes several proteins that stay statically positioned during the image acquisition (in case of the time lapse 500 ms), we wished to investigate how many RarA molecules are freely diffusing and how many are statically bound to DNA.

### Single molecule tracking of RarA-mVenus reveals that its dynamics are influenced by DNA damage

We employed single molecule fluorescence microscopy and automated single molecule tracking (SMT)^39^ to analyse RarA dynamics at the single molecule levels and in real time, using three conditions: unperturbed exponential growth, treatment with H_2_O_2_ (0.5 mM) or with MMC (50 ng/ml). A 60 min exposure to the drugs was considered as the best condition for the analysis of the mobility response, as the maximum plateau concerning focus formation was reached for every mutant in every condition at this time in the epifluorescence screening (see Fig. 2).

There are two principle ways to perform SMT: a) using stochastic photoactivation of e.g. PAmCherry fusions, in which continuous (weak) 408 nm illumination induces red fluorescence of the FP, which is tracked by continuous strong 561 nm laser illumination until the molecule bleaches and another molecule is switched on, or b) using YFP tracking, in which YFP (in our case mVenus) molecules are imaged with a continuous 514 nm laser, which bleaches fluorophores until few molecules remain that can be tracked. Usually, the first 500 to 1000 frames from the stream acquisition (maximum of 5000 frames) are discarded because cells contain more than single FPs. Single molecules can be identified because they bleach in one characteristic single step. We are using YFP SMT because in our hand, *B*. *subtilis* cells stop growing when excited with light of less than 480 nm, and because wild type cells show a relatively high background when excited with strong 561 nm light, but much less when 514 nm light is used. Cells continue to grow after imaging, showing that they can handle the phototoxicity that is generated. Please see^15,37^ and the Methods section for details on the tracking and analysis procedure.

**Figure 3:**
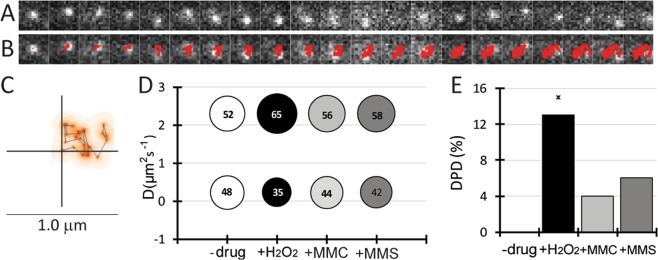
Dynamic behaviour of RarA-mVenus. (**A**) Single RarA-mVenus molecule moving in the cell (top) and the automatic detection of U-track (down). Exposure time was 10 ms. (**B**) Representation of the molecule showed in *A* in a heat map. Red colour indicates intensity of the signal. (**C**) Apparent diffusion coefficient and weight of populations for RarA-mVenus in the wild type background in exponential growth and 60 min after the addition of 0.5 mM H_2_O_2_, 50 ng/ml MMC or 5 mM MMS. Surface of the circles indicates % of molecules. (**D**) Comparison of dynamic population difference (DPD) in the different conditions. Significant differences were only seen upon H_2_O_2_ treatment.

When single RarA-mVenus molecules where observed in single-molecule microscopy, we observed two major modes of movement: rapid random movement through the cell, and slow movement around a point. Both types of movement could be found for the same molecule, as the example given Fig. 3A. Figure 3B represents the heat map of the molecule, and shows that initial long steps are followed by short steps, and ensuing longer steps. Thus, RarA molecules could be seen to alternate between random movement and confined motion around one point, i.e. a binding event.

SMT analysis of RarA-mVenus revealed the presence of two populations of molecules considering their apparent diffusion coefficient (D): a dynamic population, freely diffusing in the cytosol (D = 2.3 μm^2^ s^−1^) and a slow-moving subpopulation, probably interacting with DNA (D = 0.25 μm^2^ s^−1^). Free diffusion of mVenus in *B*. *subtilis* occurs at about 3 μm^2^ s^−1^^40^, while a large protein such as SMC (270 kDa as a dimer) moves through the DNA with 0.45 μm^2^ s^−1^^41^. Different patterns of movement (i.e. preferential locations of fast and slow molecules within the cell) will be described below. Apparent diffusion coefficients were similar in the different backgrounds studied, as expected for the same protein (Table 1, Fig. S3A), but we found considerable changes in population sizes depending on the background and the kind of DNA damage that was induced. To compare different backgrounds and conditions we defined a parameter, Dynamic Population Difference (DPD) that compares the weight on the dynamic population for one condition, and its effect on the same D value. In other words, DPD reflects the changes in the number of dynamic molecules, which are inverse for the static population.

**Table 1:** SMT analysis data for RarA-mVenus in recombination and Y-polymerases defective backgrounds.

Background	D (μm^2^ s^−1^)		−drug		+H_2_O_2_		+MMC	
	static	dyn^a^	static	dyn	static	dyn	static	dyn
Wild type	0.23	2.3	48	52	35	65	44	56
*∆recO*	0.39	2.3	61	39	47	53	57	43
*recF15*	0.2	2.1	42	58	51	49	48	52
*∆recD2*	0.26	2.2	63	37	50	50	36	64
*∆recX*	0.26	2.4	54	46	40	60	46	54
*∆addAB*	0.24	2.2	55	45	55	45	76	24
*∆recJ*	0.21	2.3	59	41	61	39	64	36
*∆recQ*	0.19	2.2	43	57	48	52	43	57
*∆recS*	0.28	2.6	71	29	50	50	56	44
*∆recU*	0.37	2.1	42	58	57	43	65	35
*∆recG*	0.21	2	46	54	43	57	55	45
*∆ruvAB*	0.26	2.7	63	37	44	56	59	41
*∆radA/sms*	0.28	2.9	57	43	57	43	58	42
*∆polY1*	0.3	2.5	52	48	33	67	34	66
*∆polY2*	0.28	2.1	47	53	38	62	48	52
DnaX-CFP	0.28	2.2	53	47	41	59	49	51

^a^dyn; dynamic. Apparent diffusion coefficients (in μm^2^ s^−1^) and static/dynamic population weights (in %) calculated by step-size distributions and Gaussian fits for RarA-mVenus in the different backgrounds studied in exponential growth, and after 60 min induction with 0.5 mM H_2_O_2_ or 50 ng/ml MMC. Cells were grown at 25 °C and images were taken at room temperature. At least 200 tracks/condition were considered for the analysis.

In wild type cells, 48% of RarA molecules were static (i.e. interacting with DNA) during unperturbed exponential growth. Note that the true number is somewhat lower, because even freely diffusing molecules can stop for very short time periods. The presence of DNA damage in all stress conditions (H_2_O_2_, MMS and MMC) produced an increase in the dynamic population (Fig. 3C), but differences were statistically significant only for H_2_O_2_ treatment (Fig. 3D). As the absence of RarA leads to a stronger phenotype after H_2_O_2_ treatment than after MMS^35^, it is reasonable to suggest that this increase of the dynamic population is influenced due to the function of RarA.

### Changes in RarA diffusion patterns in several mutant backgrounds

**Figure 4:**
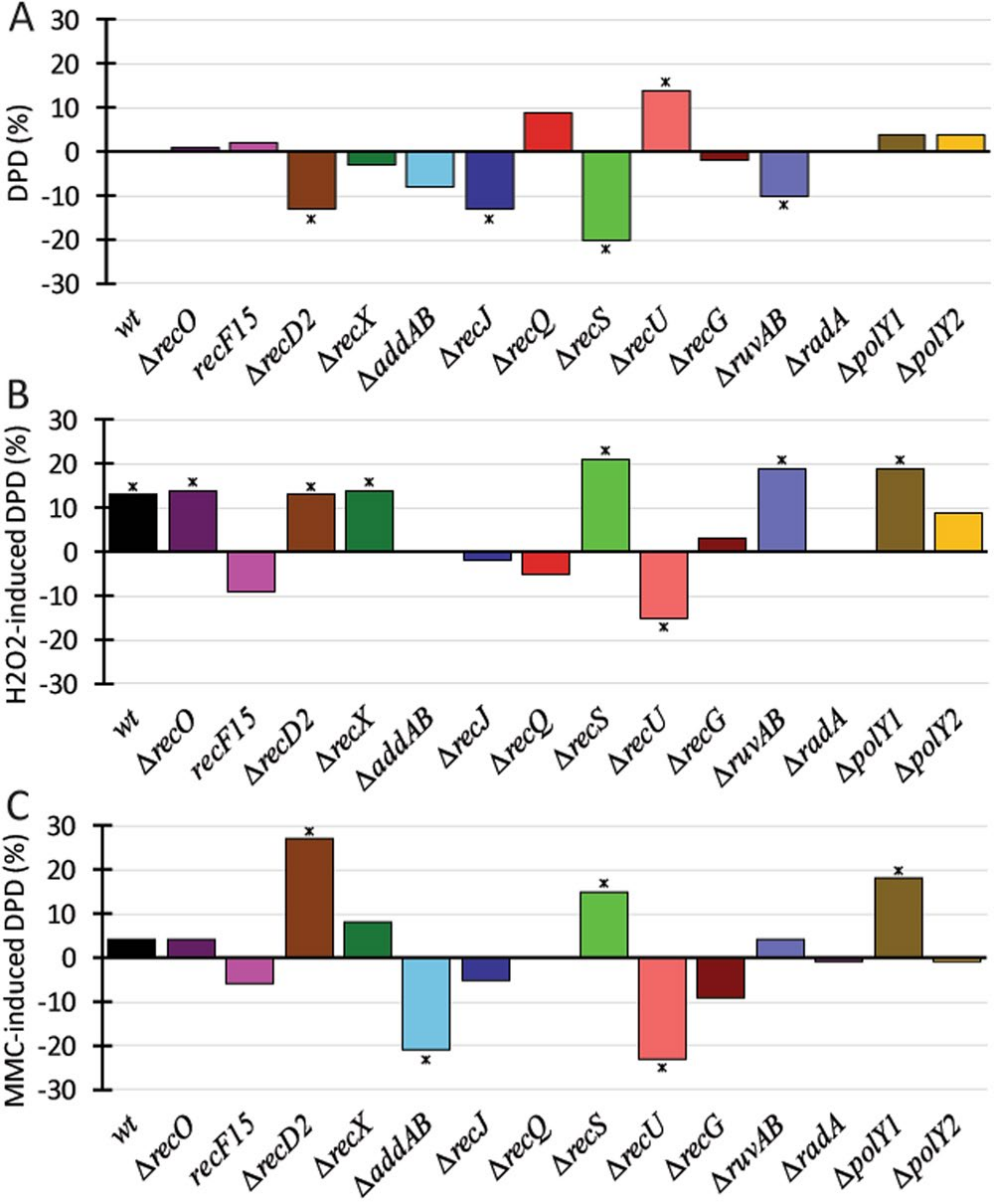
Single molecule tracking analysis for RarA-YFP in recombinational and Y-polymerase mutant backgrounds. (**A**) RarA-mVenus dynamic population difference in the backgrounds studied compared to wild type in no-drug condition. (**B**) RarA-mVenus DPDs after addition of 0.5 mM H_2_O_2_, or **C**) of 50 ng/ml MMC, for 60 min, compared to drug-free conditions in each background. * represent statistical significance in Z-test (included in SMTracker, see methods).

We next compared the differences in diffusion patterns of wild type cells growing exponentially, after exposure to H_2_O_2_ or to MMC, compared to mutant backgrounds. Between 50 and 200 cells were analysed, yielding at least 500 tracks, for each condition (i.e. background and treatment). In order to represent the differences graphically, we used the percentage and relative apparent diffusion constants and of the dynamic fraction (the static fraction making the opposite change), always in relation to the wild type, and the changes wild type cells show to DNA damage. For example, while during exponential growth, RarA-mVenus molecules are more dynamic (and thus less static/bound to DNA) during exponential growth in the absence of RecD2, a lot more molecules become dynamic in response to MMC in this mutant background (Fig. 4).

Interestingly, RarA-mVenus dynamics were clearly altered in the absence of several HR proteins. In the ∆*recD2*, ∆*recJ*, ∆*recS* and ∆*ruvAB* mutant backgrounds the dynamic population was reduced, whereas it increased in the *∆recU* cells growing exponentially (Fig. 4A). Please note that the changes shown in Fig. 4A not only incorporate the differences between wild type and mutant strains considering percentage of static/dynamic fractions (Table 1), but also incorporate changes in the diffusion rates of the dynamic fraction. In other words, not only do RarA-mVenus molecules become more dynamic in many cases, but the apparent diffusion constants also differ, which can arise from differences in the transitions between static and dynamic movement.

The response of RarA-mVenus dynamics to H_2_O_2_ damage in wild type cells included a significant increase in the dynamic population (Fig. 3D). Interestingly, RarA showed a significant difference in its dynamics in response to H_2_O_2_ treatment in the ∆*recO*, ∆*recD*2, or ∆*recX* backgrounds (compared to the behaviour in wild type cells) (Fig. 4B). Likewise, in ∆*recS*, ∆*ruvAB* and ∆*polY1* mutant cells the dynamic population was significantly increased, whereas it was significantly decreased in the ∆*recU* mutant cells (Fig. 4B). Therefore, RarA molecules became more dynamic or more static in mutant backgrounds, or did not change their binding/diffusion pattern, suggesting that there are distinct changes due to the absence of particular DNA repair proteins.

Upon MMC treatment, wild type cells did not show significant alterations in the dynamics of RarA (Fig. 3D). However, lack of RecD2, RecS or of PolY1 significantly increased the dynamic population of RarA, while absence of AddAB and RecU significantly decreased the dynamic population (Fig. 4C). Our data revealed that RarA movement is clearly altered in the absence of end-resection proteins (AddAB, RecJ, RecS and RecQ) or of Holliday junction-processing enzymes (RecU, RuvAB, RecG and RadA/Sms), while the Y-polymerases modified the response to either H_2_O_2_ (PolY2) or MMC (PolY1). RarA dynamics in cells lacking RecA mediators and modulators was dependent of the DNA damaging agents tested. Interestingly, many of these findings corroborate with a recent study of the genetic interactions of RarA^35^, showing that our tracking analysis produced reliable and interesting connections between RarA and proteins involved in HR.

In addition to the changes in dynamics, the preferential location of RarA molecules was studied by the generation of heat maps in cells normalized to a size of 3 × 1 μm. For that purpose, cells were sorted into three cell fractions: small, medium and big. This approach produced homogeneity in the cell population prior to the normalization (Fig. S3B), and thus allowed us a more accurate comparison of the subcellular distribution of molecules between different conditions and different mutants.

For exponential growth conditions in wild type cells (Fig. S3C), RarA-molecules were found throughout the cytosol with a slight preference for the one- and three-quarter positions of the cell. Please note that RarA localization is very different from that of a freely diffusing enzyme or of membrane-associated proteins, whose tracks extend until the cell border, or are within the cell membrane see^42^. RarA is mostly confined to the centre of the cell containing the nucleoid(s), similar to what was found for RecN^15^, probably because it mostly interacts with DNA in a non-specific manner. After H_2_O_2_ addition, this distribution was somewhat changed, with RarA being more homogeneously spread out on the nucleoids, and less protein being concentrated, here more towards the middle of the cell. In contrast, MMC did not change RarA distribution (Fig. S3C). This behaviour correlates with a significant increase in the dynamic population after H_2_O_2_ stress in wild type cells.

### RarA location and dynamics are related to the replication machinery

We wondered if changes in RarA dynamics might also be related to effects occurring at the DNA replication forks. Therefore, we investigated RarA-YFP in the *dnaB*37ts or *dnaC*30ts context, because a clear phenotype was observed in these mutant backgrounds when combined with ∆*rarA*^35^. Note that *B*. *subtilis* DnaB is part of the pre-primosome/helicase loader complex, and DnaC is the hexameric replicative DNA helicase. As these genes are essential, a thermosensitive mutant strategy was followed. All fluorescence analyses were performed using 25 °C as permissive temperature and 42 °C as non-permissive temperature.

**Figure 5:**
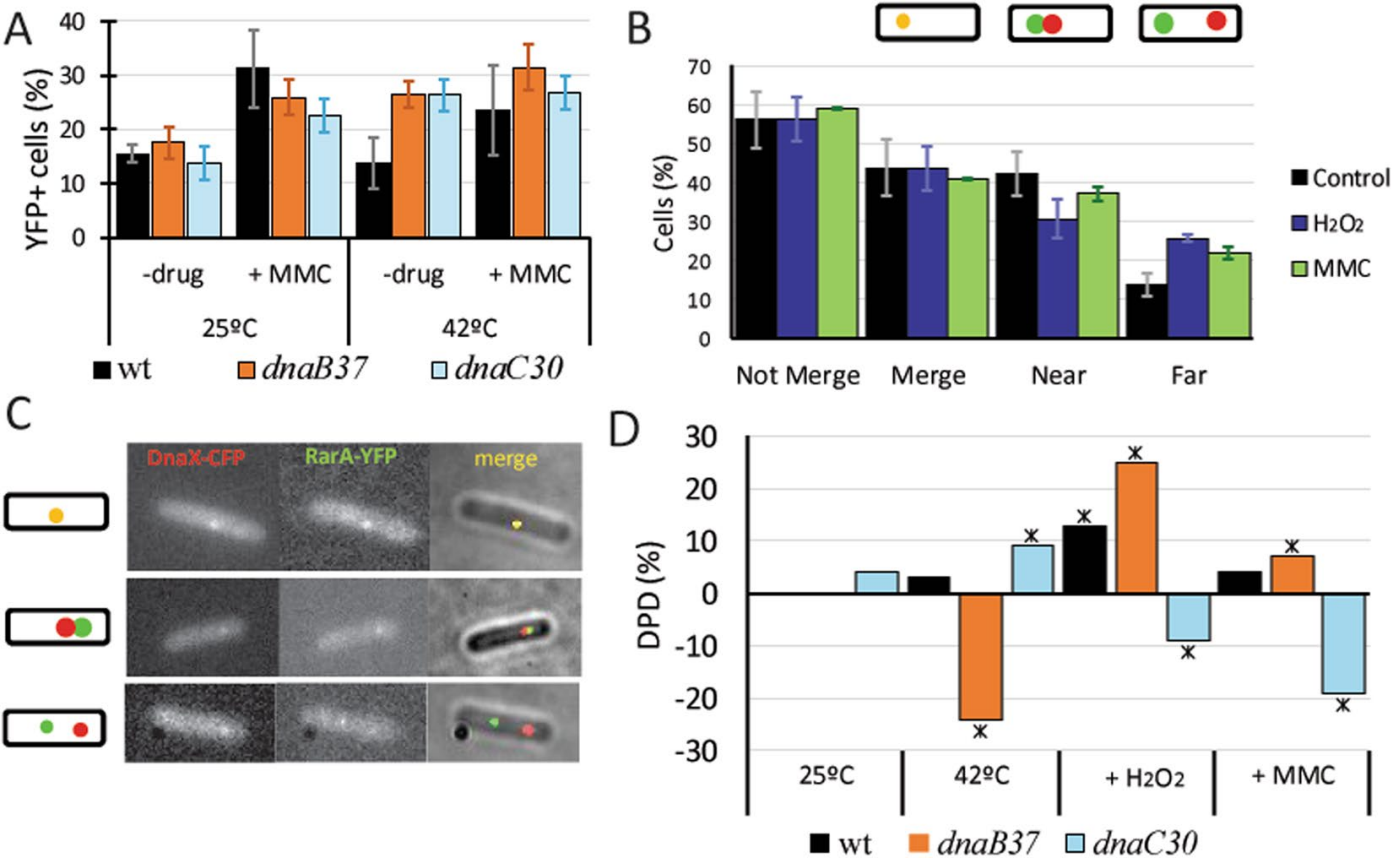
RarA interactions with components of the replication machinery. (**A**) Percentage of cells that contains RarA-YFP foci in thermosensitive replication mutants compared to wild type cells in epifluorescence microscopy, during exponential growth at 25 °C (OD_600_ = ~0.3) and after addition of MMC (50 ng /ml) for 60 min or swift to non-permissive conditions (42 °C). Error bars shows standard deviation of at least three independent experiments; (**B**) Weight of different DnaX-CFP and RarA-YFP colocalization patterns after addition of H_2_O_2_ (1 mM) or MMC (50 ng/ml) for 60 min compared to the control without drug. The percentage of not-merged foci is split at the right of the panel into two different localization patterns: near and far. (**C**) Examples of the colocalization of DnaX-CFP and RarA-YFP (up) and non-colocalizing patterns defined as near (medium) and far (down). (**D**) RarA-mVenus single-molecule DPD in thermosensitive mutants. Swift to non-permissive temperature (42 °C) leads to drastic changes in dynamics in absence of external DNA damage, and alters the normal response of RarA after addition of H_2_O_2_ or MMC.

In the first epifluorescence screening, under permissive conditions, RarA focus formation was similar in both mutant backgrounds compared with the wild type strain, before and 60 min after the addition of MMC (50 ng/ml) (Fig. 5A). Interestingly, after thermal inactivation of DnaB or DnaC, RarA focus formation increased to levels that were similar to the induction of MMC, while wild type cells were not affected by the shift to the higher temperature (Fig. 5A).

To study if the RarA foci observed upon inactivation of DnaB or DnaC are related to the replication factory (i.e. a stalled fork), a new strain was constructed by adding a DnaX-CFP construct to the RarA-YFP background. DnaX-CFP (also termed τ-CFP) is a good marker for the replication machinery, as it is part of the clamp loader complex (DnaX-HolA-HolB or τδδ´). Under exponential growth conditions, ~44% of the RarA-YFP foci co-localised with DnaX-CFP foci, and 56% did not; this number did not change when cells were exposed to H_2_O_2_ or MMC (Fig. 5B). The non-colocalizing RarA-YFP foci split into ~44% being close to the replication machinery (i.e. directly adjacent) (Fig. 5C, medium panel; Fig. 5B), and ~12% being far from the DnaX-CFP foci (i.e. at least 2 pixels distance, corresponding to 212 nm) (Fig. 5C, down panel; Fig. 5B). Interestingly, the values for the far foci changed (to the expense of the merging or close foci) considerably in the presence of DNA damage, increasing by 12%, 60 min after the addition of H_2_O_2_ or by 8% for MMC (Fig. 5B). This change in localization away from forks correlates to the changes seen in the heat maps between exponential growth and during DNA repair (Fig. S3C).

**Figure 6:**
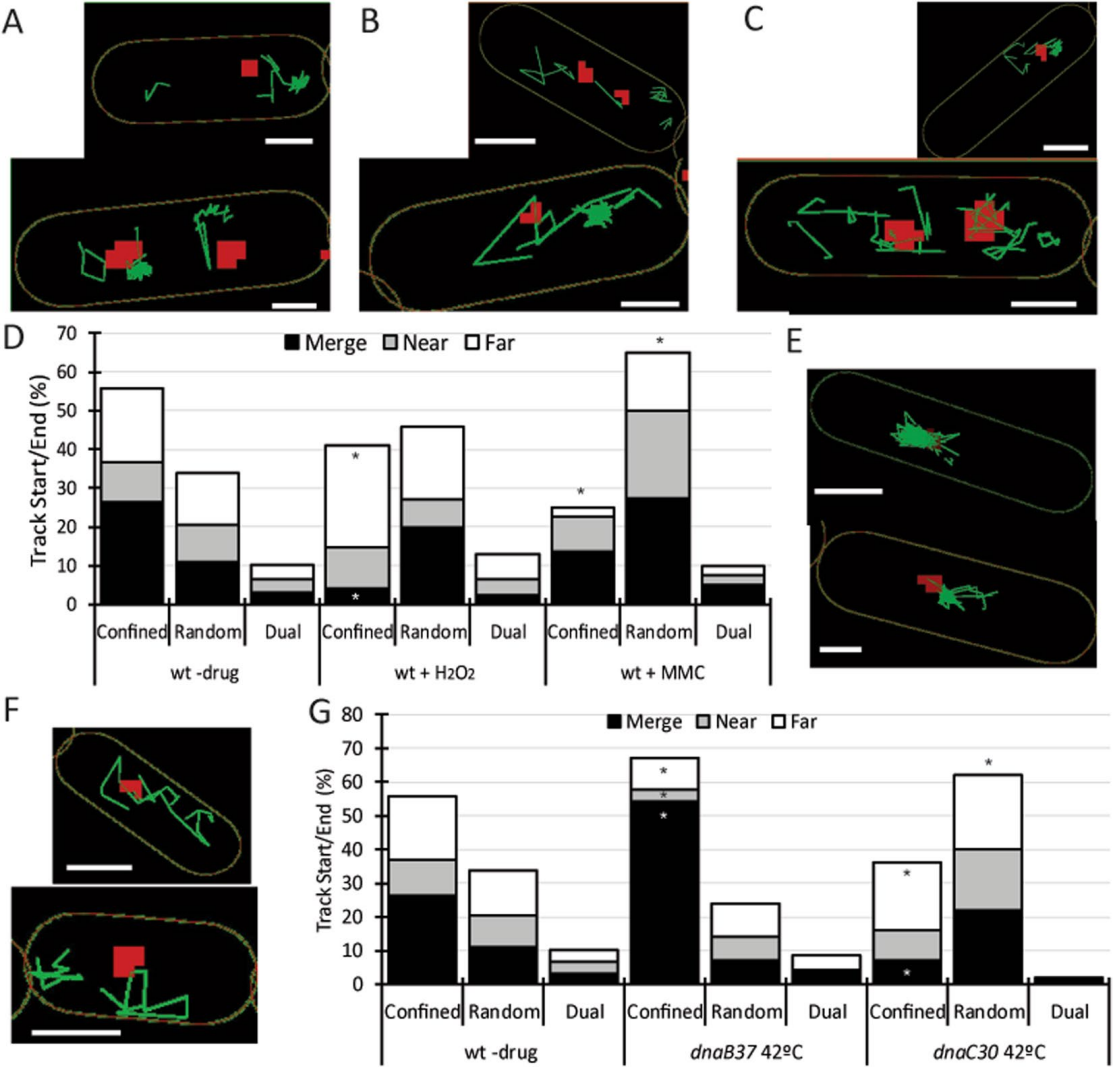
RarA interactions with the replisome. (**A–C** and **E**,**F**) Examples of cells with the distribution of RarA-mVenus tracks (green) in relation to the active replication fork, marked as DnaX-CFP (red), (**A**) in wild type cells with one or two replisomes and in the absence of DNA damage, (**B**) in the presence of H_2_O_2_ (0.5 mM, or (**C**) of MMC (50 ng/ml, for 60 min. (**E**,**F**) RarA distribution when DNA replication is halted by shift at non-permissive temperature (42 °C) in the *dnaC*30 (**E**) and *dnaB*37 (**F**) mutants. After shift to non-permissive temperature, only one DnaX focus/cell is observed. (**D**,**G**) Distribution of the weights of colocalization of the origin and end points of the RarA-mVenus tracks sorted by its movement with DnaX-CFP in exponential growth, H_2_O_2_ (0.5 mM) and MMC (50 ng/ml) (**D**) in *dnaB*37 and *dnaC*30 mutant at non-permissive temperatures (**G**). * means significant differences in Marasculio test. Scale bars correspond to 1 μm.

To further characterize the colocalization and dissociation from replication forks, SMT was done in parallel with epifluorescence. This is possible as the replication machinery movement is in a different time-scale than the tracking: it takes several minutes for the forks to change their positioning^43^, so a single DnaX-CFP image is valid for the 20 seconds of exposure to the laser for SMT. DnaX-CFP was not interfering with RarA-mVenus movement as the D values and population weights were similar to those of cells lacking DnaX-CFP (Table 1) and there are no differences when DPDs are considered (data not shown). Although RarA-mVenus tracks were located all over the cell, there was a concentration around the replication machinery in a cloud-like manner (Fig. 6A–C), and in these “clouds”, molecule tracks appear to be confined (in a manner we cannot yet explain), whereas for tracks that were not related to these clouds, also long steps of movement could be observed. To verify these observations, we measured the distance between the signal for the visible replication fork(s) and the origin and end point of each track, providing us with the estimation of localization of the tracks compared to replication forks, with the minimum diameter of a circle that contains every point of the tracks of a single molecule; this yields information about the movement of each molecule. Further, tracks were sorted by the size of the circle compared to the average size of DnaX-CFP foci (i.e. 250 nm in diameter) into three types: a) confined, when it was smaller than or equal to 250 nm; b) random, when it was bigger than 250 nm; and c) dual, in the special case that more than half of the steps of an otherwise random walk were confined (less than 250 nm) close to the forks.

In the absence of drugs (Fig. 6A), tracks were preferentially confined (~55%) and in these confined tracks, half of the track origin and ends were located merging with the replication machinery (Fig. 6D). After H_2_O_2_ exposure, confined RarA-mVenus tracks were located preferentially far from replication machinery instead of events of merging (Fig. 6B). Finally, MMC induction markedly increased the percentage of random tracks (Fig. 6C). These data suggest that during H_2_O_2_ stress, RarA leaves replication forks more frequently and binds to other places, away from forks, on the nucleoids, where likely repair events independent of replication forks take place.

### Blocking of DnaB or DnaC leads to opposing effects on the dynamics of RarA

A temperature shift, leading to non-functional DnaB37, blocks re-initiation of DNA replication rather than halting replication fork progression. Upon inactivation of DnaB, there was a marked decrease in the dynamic population of RarA (Fig. 5D, Table 2). On the other hand, stalling of DNA replication progression via thermal inactivation of DnaC led to an increase in the dynamic population of RarA. We compared the changes in the localization of RarA tracks relative to replication forks. After temperature shifts, RarA tracks in *dnaB*37*ts* cells (Fig. 6F) were mostly confined (~70%), and specially merged with the replication machinery (54%) (Fig. 6G), while tracks in *dnaC*30*ts* cells (Fig. 6E), mostly presented random movement (~60%), and confined tracks were located preferentially in “far” positions relative to replication forks (Fig. 6G). Taken together, these data suggest that DnaC contributes to RarA binding to the replication machinery while a putative DnaB interaction relates to the removal of RarA from a newly assembled replication fork, which does not occur in the absence of DnaB.

**Table 2:** SMM data for replication thermosensitive mutants.

Background	D (μm^2^ s^−1)^	25 °C −drug	42 °C −drug	42 °C+H_2_O_2_	42 °C+MMC
	static dyn^a^	static dyn	static dyn	static dyn	static dyn
Wild type	0.23 2.3	48 52	45 55	ND ND	ND ND
*dnaB*37	0.2 1.8	41 59	65 35	40 60	58 42
*dnaC*30	0.19 2.0	38 62	29 71	38 62	48 52

Apparent diffusion coefficient (in μm^2^ s^−1^) and population weights (in %) calculated by step-size distribution and Gaussian fit for RarA-mVenus in the replication deficient thermosensitive mutants studied in exponential growth for 60 min at permissive (25 °C) and non-permissive (42 °C) temperature in the presence or absence of H_2_O_2_ (0.5 mM) or MMC (50 ng/ml). At least 200 tracks/condition were considered for the analysis. ^a^dyn, dynamic; ND, not done.

When either H_2_O_2_ or MMC were added under semi-permissive temperatures, the dynamic RarA population was significantly increased in *dnaB*37*ts* cells, while in the *dnaC*30*ts* strain, it became significantly more static (Fig. 5D), strongly supporting the idea of opposing effects of DnaB and DnaC to RarA recruitment at replication forks.

## Discussion

Our work shows that the widely conserved protein RarA, a AAA+ type ATPase of poorly understood function, plays a role in the cellular response to the induction of DNA damage, in close connection to proteins involved in replication fork re-start (DnaB and DnaC), and to several proteins that affect RecA activities, either positively (RecO, RecF) or negatively (RecU, RecX, RecD2). We also show that single molecule tracking provides a valuable tool for a screen for protein interactions, or more generally for protein connectivity, and that proteins can be tracked relative to defined positions within the cell.

Previously, RarA has been linked to the replication machinery in *E*. *coli*, and has even used as a marker for replication forks^24,31^. Using epifluorescence and single-molecule microscopy we show that for *B*. *subtilis*, this is not always the case, as ~20% of the foci found were not related to the replication fork(s). Moreover, by tracking single molecules, we can see that about 50% of RarA molecules are statically associated with DNA (and a considerable fraction of these at DNA sites other than the forks), while 50% move throughout the cells. Interestingly, after induction of damage by H_2_O_2_, the fraction of freely moving RarA molecules increased significantly, and also the number of molecules with confined movement (i.e. bound to DNA) far from the replication forks. These data suggest that while RarA can be recruited to stalled or collapsed forks, it is also recruited to DNA damage (i.e. broken DNA ends) at different places on the chromosome, independently of the replication machinery^8,44–47^.

The location of RarA at the replication machinery during exponential growth can be well explained considering its known interaction with SsbA^28,31^. DnaC, which translocates on the lagging-strand template in a 5′ **→** 3**′** direction, is the hexameric replicative helicase that is part of the primosome, which initiates the assembly of the replisome^48^. Interestingly, when *dnaC*30*ts* mutants were exposed to non-permissive temperatures, RarA movement became significantly more dynamic, meaning that it was lost from the forks. Contrarily, the opposite effect occurred in *dnaB37* cells. DnaB is a pre-primosome component involved in DnaA-dependent initiation or PriA-dependent re-initiation of DNA replication by contributing to loading of DnaC. After this, DnaB dissociates from the (re)initiation complex^48–52^. Localisation of the tracks showed that RarA molecules confined to the replication forks are more abundant when DnaB is non-functional (Fig. 6E,F). Altogether, these data suggest that DnaC contributes to the recruitment of RarA to, whereas DnaB contributes to the removal from replication forks. It is also possible that RarA works prior to DnaB, contributing to the control of pre-primosome assembly, and might leave the initiation complex together with all pre-primosome components (PriA, DnaB, DnaD and DnaI)^28^. This connection seems to be an important role of RarA during replication, because its absence in both *dnaB* and *dnaC* backgrounds was reported to lead to a loss of viability in semi-permissive conditions^35^. The importance of RarA, in combination with other recombination factors, to correct and repair DNA damage during replication is confirmed by the strong reduction in viability under normal growth of the Δ*recA* Δ*rarA* and Δ*recO* Δ*rarA* double mutant, and by the genetic interactions of RarA with RecA, with RecA mediators (RecO, RecR) and modulators (RecF, RecX, RecU)^35^.

Our SMT approach also revealed a strong influence on the dynamics of RarA in the absence of other recombination factors, such as RecJ, RecS, AddAB, or RuvAB. Interestingly, in all these backgrounds, a deletion of *rarA* partially suppresses the sensitivity to DNA damaging agents^35^. All of the proteins mentioned above have been characterized in the context of replication fork regression and replication fork reactivation in previous studies^53–55^, including RarA for both prokaryotes^22,29^ and eukaryotes^32^, but they are also implicated in the formation of DNA repair centres^14^. Once RarA is recruited, its main function seems to be related to RecA and its regulators, as we found that the genetic and dynamic interactions with RecO and RecF differ from those with RecX. Microscopy observations revealed an opposite behaviour of RarA in the *∆recO* or *recF15* context compared to the *∆recX* strain, as foci formation observed in the latter was decreased while in the other two it was enhanced compared to wild type cells, and additionally, it occurred earlier. This may be related to the formation of RecA-ssDNA nucleoprotein filaments, which is facilitated by RecF and RecO, and inhibited by RecX and RecU^17,20,21^. However, dynamics of RarA were also affected in an opposite manner between *recX* and *recU* mutant backgrounds, so the interpretation of the changes seen in RarA dynamics are rather intricate. SMT experiments also pointed out that RecF may be an important factor for RarA mobilization in the response to H_2_O_2_ (Fig. 4B).

Observation by epifluorescence, and especially with SMT, revealed differences in RarA mobilization in the presence of DNA damage (Figs 2, 3), which correlates with the genetic data showing that RarA is involved in DNA damage repair after addition of H_2_O_2_, but not when cells are exposed to MMS or MMC see Fig. 1A and^35^. H_2_O_2_-induced DNA damage lead to an increase in the dynamic population of RarA (Fig. 4B), but also to the recruitment of RarA to areas located out of the influence of the replication forks. This mobilization is influenced by many recombination factors, like RecQ, RecJ, RecF, RecU and RecG (Fig. 4B). It is possible that the inhibition of the mobilization of RarA in the absence of long-range end resection (as defined by *ΔaddAB*, *ΔrecQ* and *ΔrecJ*) is due to the ssDNA platform needed for the recruitment of SsbA^31^. Interestingly, we have found a different behaviour of RarA with regard to the absence of RecQ or RecS. *B*. *subtilis* RecS (56.5 kDa) shares 36% identity with RecQ (67.3 kDa)^56^, and both proteins are required for RecJ activity^16^. RarA becomes significantly more static in the *ΔrecS* context, but becomes more mobile upon DNA damage. In contrast, the opposite effect occurred in the absence of RecQ, RarA became significantly more dynamic in the absence of damage, but more static upon H_2_O_2_ stress. Thus, our SMT analysis has allowed us to identify functional differences between both RecQ-like DNA helicases for *B*. *subtilis* cells. In humans, it is known that WRNIP1/WHIP physically interacts with WRN, a RecQ-like helicase. We predict that RarA interacts with both RecS and RecQ via SsbA^31^.

SMT analysis also revealed an interaction with PolY1, indicating that RarA could play a role in MMC-mediated mutagenesis by translesion synthesis, for which PolY1 is needed^57^, and thus this function could be conserved in evolution, as yeast Mgs1 is known to physically interact with DNA polymerase δ and proposed to regulate its processivity and fidelity^32^.

Taken together, our data suggest a dual role for RarA, in replication-related repair and in non-replication-related DNA repair (formation of DNA repair centres), which is consistent with but also different from the data obtained in *E*. *coli*^27,29^. They support the idea of DNA repair centres being formed outside of the replication forks in *B*. *subtilis*^14^, rather than the absolute need of the presence of a stalled replication fork for homologous recombination^2^. As explained above, the role of RarA in replication seems to be related to interactions with pre-primosome components directly, or indirectly, via SsbA and PriA^28^. We suggest that RarA is a major factor for regulating pre-primosomal activity outside *oriC*, which depends on PriA-dependent replication re-initiation^28^ or in a more complex pathway implicating RecG, RuvAB and/or RecU for replication restart at a collapsed replication fork^44^. According to our study using SMT, RarA’s role in replication might be also be regulated by a lack of end resection (RecS, RecJ and AddAB) and by RecD2 or RuvAB, whose absence strongly affects RarA mobility in exponentially growing cells.

## Supplementary information

Supplementary Dataset 1

movie S1

## Data Availability

All data generated or analysed during this study are included in this published article and its Supplementary Information files.
