# Supplementary material for: Single molecule tracking reveals functions for RarA at replication forks but also independently from replication during DNA repair in Bacillus subtilis
Source: Sci Rep. 2019 Feb 13;9:1997. doi: 10.1038/s41598-018-38289-6 (PMC6374455; doi:10.1038/s41598-018-38289-6)
Supplement: Supplementary file 1 — Supplementary Dataset 1 [file 41598_2018_38289_MOESM1_ESM.pdf]

Supplementary Material

Single molecule tracking reveals functions for RarA at replication forks but also independently from replication during DNA repair in *Bacillus subtilis*

Hector Romero[1,2,3], Thomas C. Rösch[1,2], Rogelio Hernández-Tamayo[1,2], Daniella Lucena[1,2], Silvia Ayora[3], Juan C. Alonso[3*], Peter L. Graumann[1,2*]

[1]SYNMIKRO, LOEWE-Zentrum für Synthetische Mikrobiologie, Hans-Meerwein-Straße, Mehrzweckgebäude, 35043 Marburg, [2]Fachbereich Chemie, Hans-Meerwein-Straße 4, 35032 Marburg, Germany, [3]Department Microbial Biotechnology, Centro Nacional de Biotecnología, CNB-CSIC, 3 Darwin St., 28049 Cantoblanco, Madrid, Spain.

Table S1A. *Bacillus subtilis* strains used

Strains	Genotype source strain	Source/parent strain	Strains	Relevant genotype	Source
BG214	+ wild type [a]		BG1067	+ Δ*rarA*	This work
BG190	+ Δ*recA*	[1]	BG1555	+ Δ*recA*, Δ*rarA*	This work
BG439	+ Δ*recO*	[2]	BG1433	+ Δ*recO*, Δ*rarA*	This work
BG129	+ *recF15*	[3]	BG1055	+ *recF15*, Δ*rarA*	This work
BG1455	+ Δ*recD2*	[4]	BG1421	+ Δ*recD2*, Δ*rarA*	This work
BG1065	+ Δ*recX*	[5]	BG1371	+ Δ*recX*, Δ*rarA*	This work
BG1337	+ Δ*addAB*	[6]	BG1107	+ Δ*addAB*, Δ*rarA*	This work
BG675	+ Δ*recJ*	[7]	BG1059	+ Δ*recJ*, Δ*rarA*	This work
BG705	+ Δ*recQ*	[7]	BG1575	+ Δ*recQ*, Δ*rarA*	This work
BG425	+ Δ*recS*	[7]	BG1563	+ Δ*recS*, Δ*rarA*	This work
BG855	+ Δ*recU*	[8]	BG1083	+ Δ*recU*, Δ*rarA*	This work
BG1131	+ Δ*recG*	[9]	BG1103	+ Δ*recG*, Δ*rarA*	This work
BG703	+ Δ*ruvAB*	[10]	BG1351	+ Δ*ruvAB*, Δ*rarA*	This work
BG1245	+ Δ*radA*	[11]	BG1373	+ Δ*radA*, Δ*rarA*	This work
BG905	+ Δ*polY1*	[12]	BG1401	+ Δ*polY1*, Δ*rarA*	This work
BG907	+ Δ*polY2*	[12]	BG1403	+ Δ*polY1*, Δ*rarA*	This work
BG193	+ *dnaB*37	[13]	BG1687	+ *dnaB*37, Δ*rarA*	This work
BG196	+ *dnaC*30	[13]	BG1681	+ *dnaC*30, Δ*rarA*	This work
BG198	+ *dnaG*20	[13]	BG1661	+ *dnaG*20, Δ*rarA*	This work
BG199	+ *dnaF*33	[13]	BG1685	+ *dnaF*33, Δ*rarA*	This work
BG201	+ *dnaX*51	[13]	BG1659	+ *dnaX*51, Δ*rarA*	This work
BG1679	+ *dnaE*58	This work	BG1683	+ *dnaE*58, Δ*rarA*	This work

[a]*trpCE metA*5 *amyE1 ytsJ*1 *rsbV*37 *xre*1 *xkd*A1 *att*$^{\mathrm{SP\beta}}$ *att*$^{\mathrm{ICE}Bs1}$

Table S1B. *B. subtilis rarA-yfp* and its mutant variants

Strains[a]	Relevant genotype	Source
BG1331	+ *rarA-yfp*	This work
PG3171	+ *rarA-mVenus*	This work
BG1445	+ *rarA-yfp*, Δ*recO*	This work
BG1345	+ *rarA-yfp*, *recF15*	This work
BG1347	+ *rarA-yfp*, Δ*recD2*	This work
BG1349	+ *rarA-yfp*, Δ*recX*	This work
PG3316	+ *rarA-yfp*, Δ*addAB*	This work
PG3423	+ *rarA-yfp*, Δ*recJ*	This work
PG3318	+ *rarA-yfp*, Δ*recQ*	This work
PG3424	+ *rarA-yfp*, Δ*recS*	This work
BG1443	+ *rarA-yfp*, Δ*recU*	This work
PG3317	+ *rarA-yfp*, Δ*recG*	This work
PG3426	+ *rarA-yfp*, Δ*ruvAB*	This work
PG3429	+ *rarA-yfp*, Δ*radA*	This work
PG3427	+ *rarA-yfp*, Δ*polY1*	This work
PG3428	+ *rarA-yfp*, Δ*polY2*	This work
PG3174	+ *rarA-yfp*, *dnaX-cfp*	This work
BG1451	+ *rarA-yfp*, *dnaB*37	This work
BG1453	+ *rarA-yfp*, *dnaC30*	This work
PG3430	+ *rarA-yfp*, *dnaB*37, *dnaX-cfp*	This work
PG3431	+ *rarA-yfp*, *dnaC30*, *dnaX-cfp*	This work

[a]All strains are derivatives of *B. subtilis* BG214 (*trpCE metA*5 *amyE1 ytsJ*1 *rsbV*37 *xre*1 *xkd*A1 *att*^{SPß} *att*$^{ICE\textit{Bs}1}$)

Movie S1: Exponentially growing cells expressing RarA-mVenus as sole source of the protein, images taken every 3 minutes. Shown are overlays of bright field and mVenus (variant of YFP) fluorescence (shown in yellow) images. 4 frames per second, White bar 5 μm. Note that the cells grow in short chains of cells.

Figure S1

A

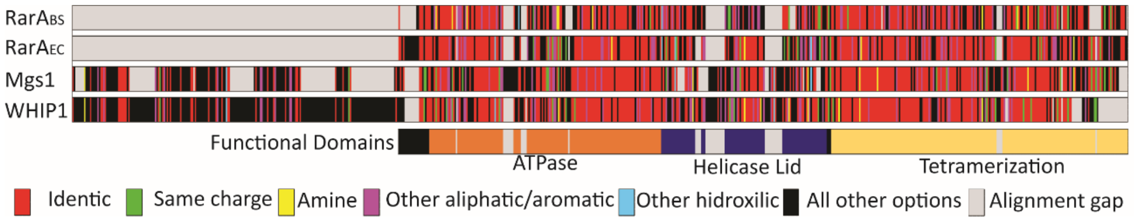

B

Species	Identity (%)			
	B. subtilis RarA	*E. coli* RarA	*S. cerevisae* Mgs1	*H. sapiens* WHIP1
B. subtilis RarA	100	35.7	38.9	38.2
E. coli RarA	35.7	100	40.3	40.6
S. cerevisae Mgs1	38.9	40.3	100	42.0
H. sapiens WHIP1	38.1	40.6	42.0	100
amino acids number	422	447	587	665

Figure S1. Comparison of RarA protein family (A) BLASTA for the sequences obtained in NCBI database and the functional domains predicted by Page *et al.* [14] for *E. coli* RarA. Color-code represent the identities or the type of minor changes (polar with same charge, green; amine, yellow; or same kind of side chain, pink and blue). Figure is scaled to the size of proteins except of the gap needed for alignment. (B) Identity values for RarA homologues in evolution. The protein increases its size in eukaryotes, the identity is conserved

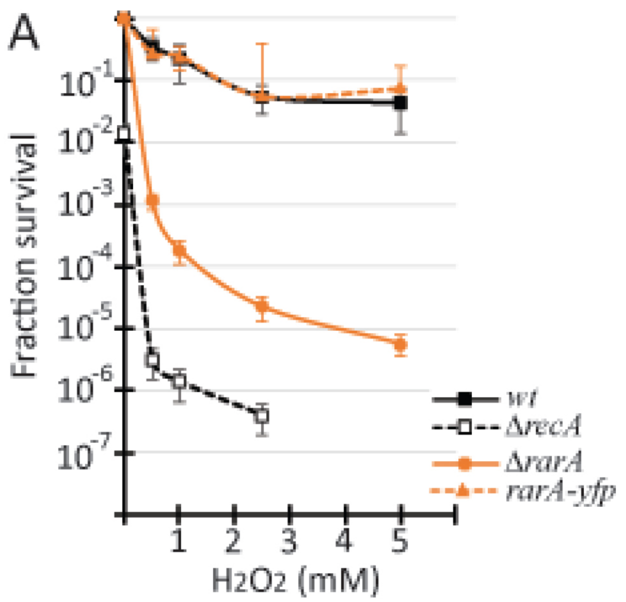

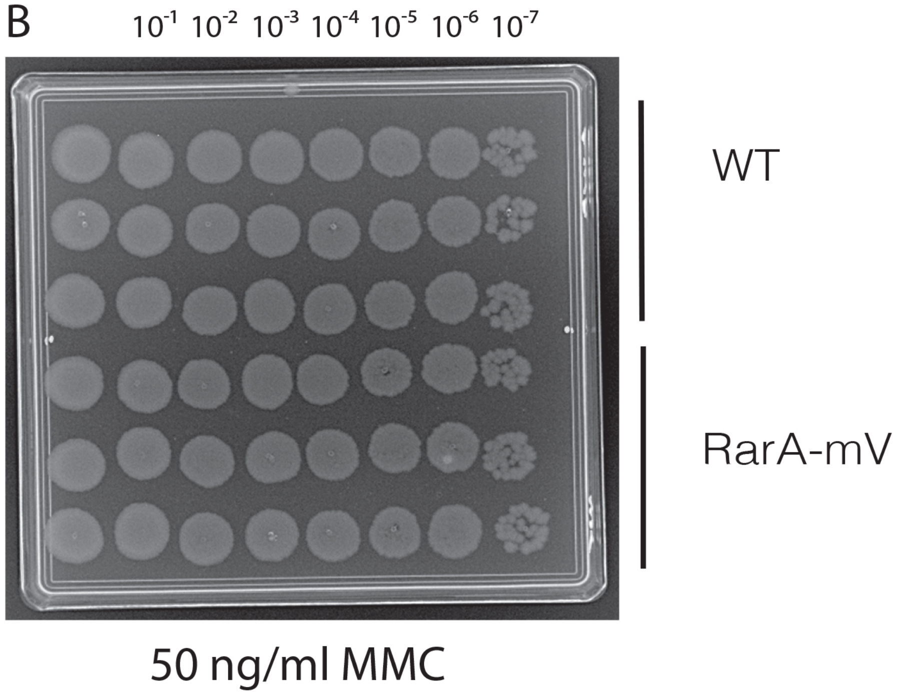

Fig. S2 Survival assays. (A) Chronic viability assay for the RarA-mVenus expressing strain. (B) Acute assay for survival of 60 minutes addition of MMC. Three independent colonies/assays are shown for wild type and for RarA-mVenus expressing cells.

Figure S3

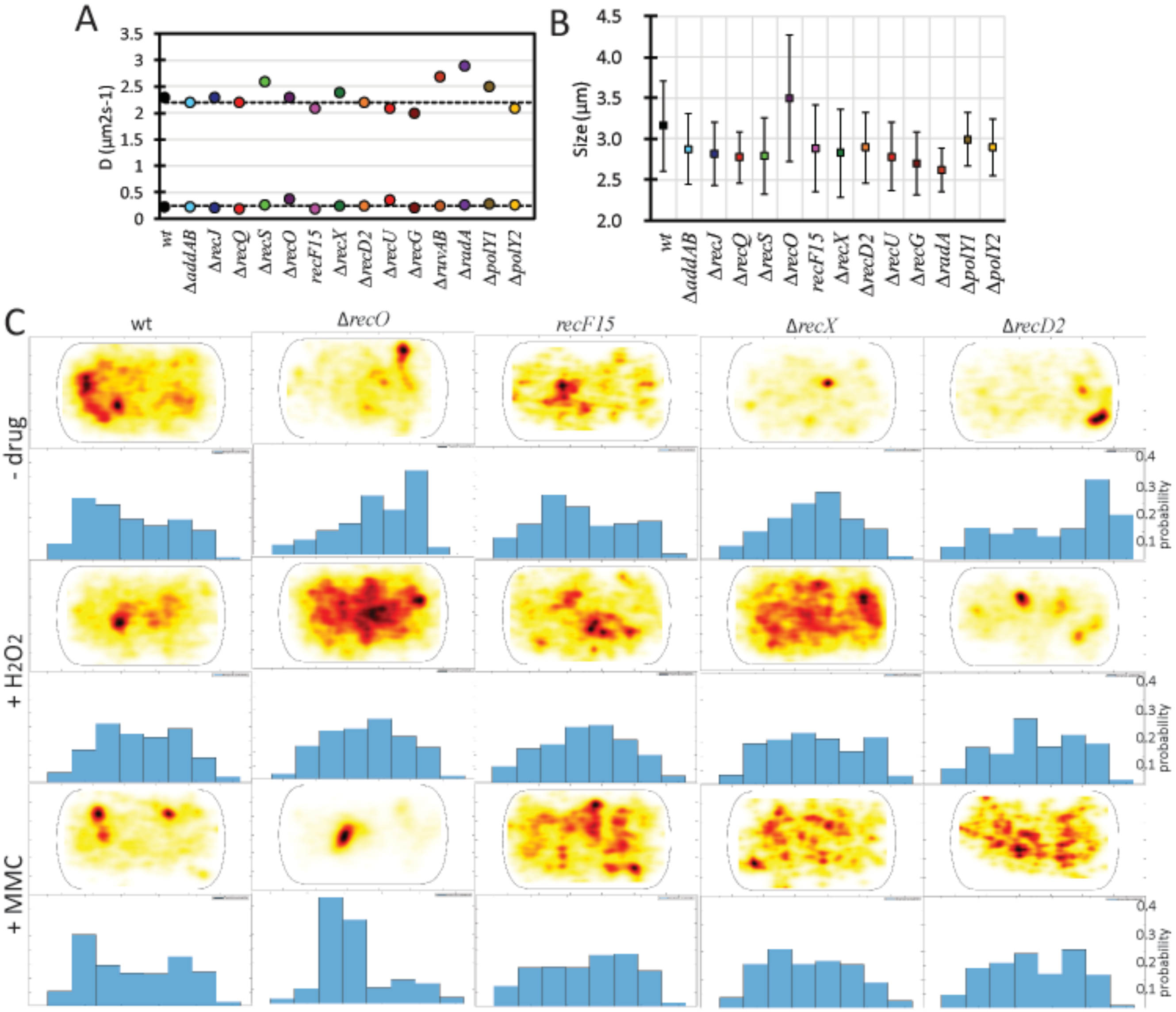

Figure S3. (A) Diffusion coefficients for static and dynamic populations calculated by Gaussian fit based in the step-size distribution. Values of each background were analysed separately, while dotted lines correspond to the analysis of all strains taken together. (B) Cell size considered prior to the normalization for the heat maps for the different backgrounds. (C) Heat maps and track distribution probability in X-axis for medium cells in the wild type, Δ*recO*, *recF15*, Δ*recX* and Δ*recD2* in absence of DNA damage, or after induction with H_2O_2 (0.5 mM) or MMC (50 ng/ml).

References

1 Ceglowski, P., Luder, G. & Alonso, J. C. Genetic analysis of recE activities in Bacillus subtilis. *Mol Gen Genet* **222**, 441-445 (1990).

2 Fernandez, S., Kobayashi, Y., Ogasawara, N. & Alonso, J. C. Analysis of the Bacillus subtilis recO gene: RecO forms part of the RecFLOR function. *Mol Gen Genet* **261**, 567-573 (1999).

3 Alonso, J. C., Tailor, R. H. & Luder, G. Characterization of recombination-deficient mutants of Bacillus subtilis. *J Bacteriol* **170**, 3001-3007 (1988).

4 Torres, R., Romero, H., Rodriguez-Cerrato, V. & Alonso, J. C. Interplay between *Bacillus subtilis* RecD2 and the RecG or RuvAB helicase in recombinational repair. *DNA Repair (Amst)* **55**, 40-46, doi:10.1016/j.dnarep.2017.05.004 (2017).

5 Cardenas, P. P. *et al.* RecX facilitates homologous recombination by modulating RecA activities. *PLoS Genet* **8**, e1003126, doi:10.1371/journal.pgen.1003126 (2012).

6 Vlasic, I. *et al.* Bacillus subtilis RecA and its accessory factors, RecF, RecO, RecR and RecX, are required for spore resistance to DNA double-strand break. *Nucleic acids research* **42**, 2295-2307, doi:10.1093/nar/gkt1194 (2014).

7 Sanchez, H., Kidane, D., Cozar, M. C., Graumann, P. L. & Alonso, J. C. Recruitment of Bacillus subtilis RecN to DNA double-strand breaks in the absence of DNA end processing. *Journal of bacteriology* **188**, 353-360 (2006).

8 Fernandez, S., Sorokin, A. & Alonso, J. C. Genetic recombination in Bacillus subtilis 168: effects of recU and recS mutations on DNA repair and homologous recombination. *J Bacteriol* **180**, 3405-3409 (1998).

9 Sanchez, H., Carrasco, B., Cozar, M. C. & Alonso, J. C. Bacillus subtilis RecG branch migration translocase is required for DNA repair and chromosomal segregation. *Mol Microbiol* **65**, 920-935, doi:MMI5835 [pii]

10.1111/j.1365-2958.2007.05835.x (2007).

10 Sanchez, H. *et al.* The RuvAB branch migration translocase and RecU Holliday junction resolvase are required for double-stranded DNA break repair in Bacillus subtilis. *Genetics* **171**, 873-883, doi:genetics.105.045906 [pii]

10.1534/genetics.105.045906 (2005).

11 Gándara, C. & Alonso, J. C. DisA and c-di-AMP act at the intersection between DNA-damage response and stress homeostasis in exponentially growing Bacillus subtilis cells. *DNA repair* **27**, 1-8, doi:10.1016/j.dnarep.2014.12.007 (2015).

12 Raguse, M. *et al.* Bacillus subtilis DisA helps to circumvent replicative stress during spore revival. *DNA repair* **59**, 57-68, doi:10.1016/j.dnarep.2017.09.006 (2017).

13 Alonso, J. C., Stiege, C. A., Tailor, R. H. & Viret, J. F. Functional analysis of the dna (Ts) mutants of Bacillus subtilis: plasmid pUB110 replication as a model system. *Mol Gen Genet* **214**, 482-489 (1988).

14 Page, A. N., George, N. P., Marceau, A. H., Cox, M. M. & Keck, J. L. Structure and biochemical activities of *Escherichia coli* MgsA. *J. Biol. Chem.* **286**, 12075-12085, doi:10.1074/jbc.M110.210187 (2011).
